# Takotsubo syndrome and atrial myxoma—identifying a new trigger: a case report

**DOI:** 10.3389/fcvm.2024.1323492

**Published:** 2024-02-13

**Authors:** Kevin Velarde-Acosta, Robert Sandoval, Luis Falcón-Quispe, William Efrain Anicama Lima, Roberto Baltodano-Arellano

**Affiliations:** ^1^ Clinical Cardiology Service, Hospital Guillermo Almenara Irigoyen – EsSalud, Lima, Peru; ^2^ Cardiac Imaging Area of Cardiology Service, Hospital Guillermo Almenara Irigoyen – EsSalud, Lima, Peru; ^3^ School of Medicine, Universidad Nacional Mayor de San Marcos, Lima, Peru; ^4^ Pathological Anatomy Service, Hospital Guillermo Almenara Irigoyen - EsSalud, Lima, Peru

**Keywords:** takotsubo syndrome, atrial myxoma, multimodal imaging, HeartTeam management, case report

## Abstract

Takotsubo syndrome (TTS) is a rare cardiomyopathy, but its prevalence is increasing due to the greater availability of diagnostic tools, whose pathophysiology is unknown; however, the evidence points to an excess of catecholamines that ends up generating cardiac stunning. The cause of excessive sympathetic discharge is multifactorial, and some tumors may be related to their origin. In this case report, we present a female patient with TTS whose only identified triggering factor was an atrial myxoma, which generated an unusual clinical presentation. Current multimodal diagnostic tools together with the multidisciplinary evaluation of the HeartTeam allowed an accurate diagnosis and an adequate management of the clinical picture.

## Introduction

Takotsubo cardiomyopathy (TTS) is a rare syndrome, with a predilection for the female sex, which usually mimics an acute coronary syndrome (ACS) and is characterized by transient ventricular systolic dysfunction (1, 2). Within its most common presentation, it exhibits akinesia-hypokinesia of the cardiac apex and medial segments, and hyperkinesia of the basal segments, with complete recovery after months (3). The pathogenesis is multifactorial, but the most likely mechanism is an excessive adrenergic discharge that generates cardiac stunning (3). Anecdotal cases of cardiac tumors, producing catecholamines, responsible for TTS, have been described (4). Among them are myxomas, the most common primary and benign cardiac tumors, typically located in the left atrium (5, 6). The association of these pathologies is extremely rare and constitutes a diagnostic and therapeutic challenge for the treating multidisciplinary team.

## Case report

A 54-year-old female patient, Peruvian Andean, was admitted to the emergency department due to very intense, non-radiating oppressive chest pain, associated with nausea, vomiting and sweating and without any apparent physical nor emotional triggering factor. The patient also reported dyspnea on moderate exertion (NYHA II). As relevant medical history, she suffered a lacunar infarction in posterior limb of the left internal capsule 3 months before admission; the patient had no other relevant personal or family pathological history. On physical examination, blood pressure was 130/77 mmHg, heart rate was 60 bpm, and SpO2 was 91% (without supplemental oxygen) so support with nasal cannula at 3 Lt/min was started, reaching an SpO2 of 96%. Skin was warm, no edema in lower limbs, capillary refill was <2 s, parvus pulse present in all 4 extremities, jugular ingurgitation was present. On auscultation, she had a low-intensity II/VI systolic murmur in the aortic focus without irradiation and fine crackles in the lower half of both lung fields. On neurological examination, the patient had mildly decreased right brachycrural muscle strength without sensory deficit or altered level of consciousness. The rest of the physical examination was normal.

## Investigation

**Figure 1:**
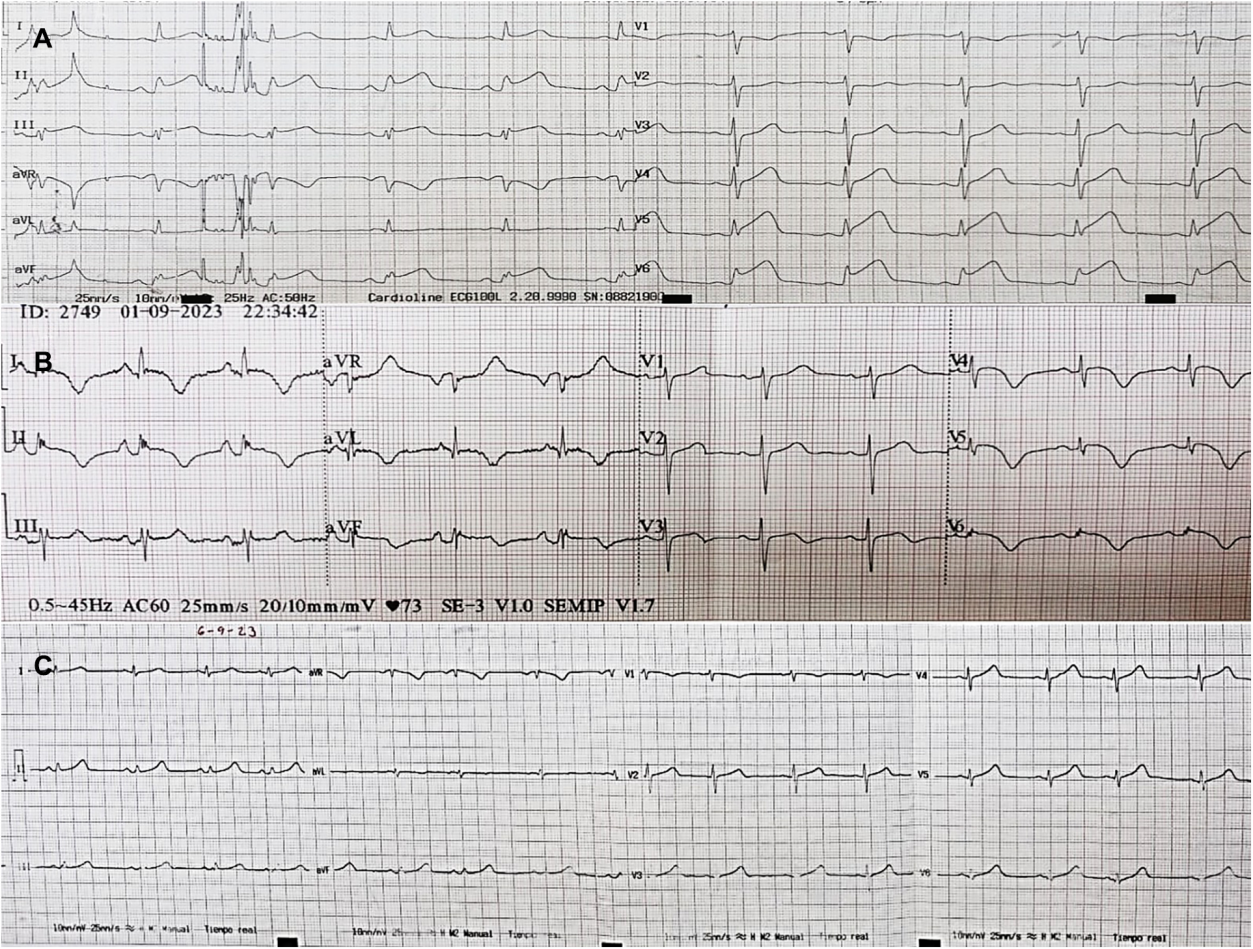
ECGs sequence. (**A**) Admission ECG: Sinus rhythm/HR 65 rpm/PR 140 ms/QRS axis +30°/QRS, 80 ms/QT 320 ms/ST segment elevation in DI, DII, DIII, AVF, V5, V6. (**B**) Day 2 ECG: Sinus rhythm/HR 70 rpm/PR 160 ms/QRS axis +30°/QRS 80 ms/QT 420 ms/negative T waves in DI, DII, aVL, aVF, V4, V5, V6/biphasic T wave V3. (**C**) Day 6 ECG: Sinus rhythm/HR 65 rpm/PR 140 ms/QRS axis +60°/QRS 80 ms/QT 400 ms/normalization of the ST segment and T waves.

**Figure 2:**
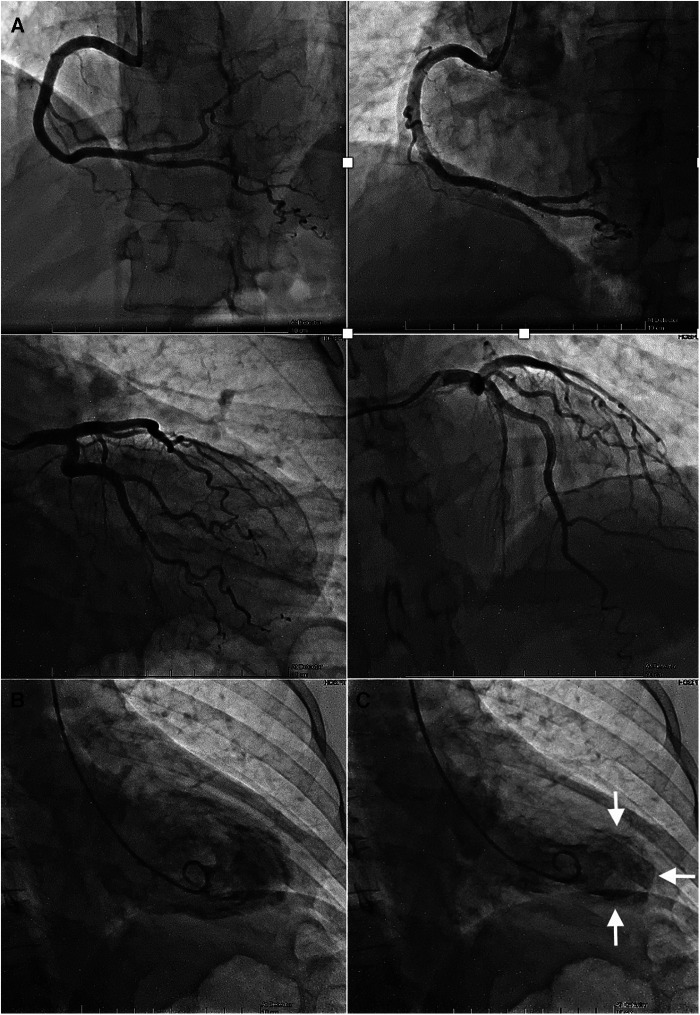
Coronary angiography and ventriculography. (**A**) Angiographically normal right and left coronary arteries. (**B**) Left ventriculography in diastole, compared to systole (**C**), where an apical balloon is observed (white arrows).

**Figure 3:**
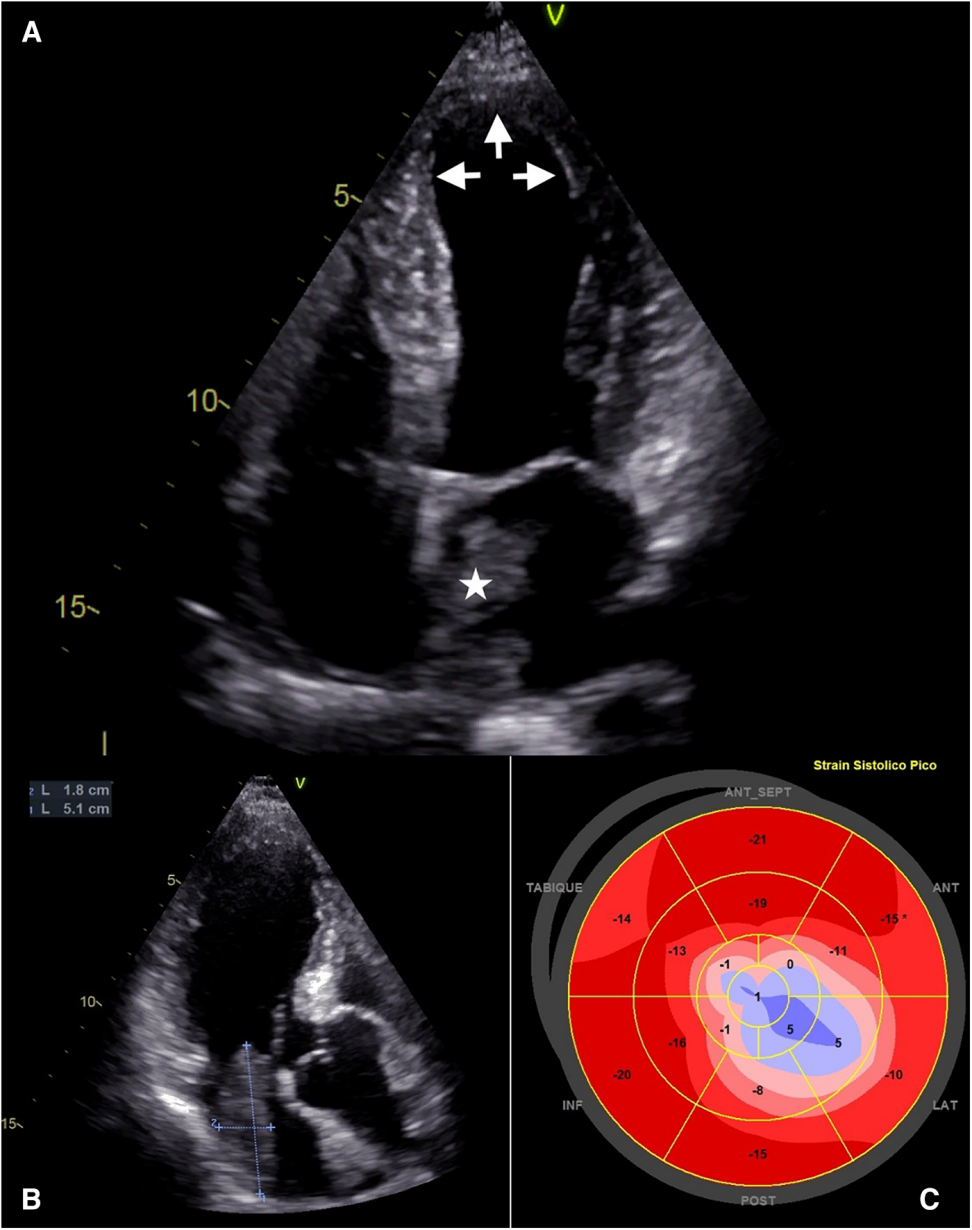
Transthoracic echocardiography. (**A**) TTE, apical 4-chamber view. Dilation of the ventricular cavity is seen at the apical level in systole (white arrows), and a mass is evident in the left atrium implanted in the interatrial septum. (**B**) TTE, apical 3-chamber view. Mass in the left atrium with a maximum diameter of 51 mm. (**C**) Polar map of the left ventricular strain. Illustration of apical-medial involvement and basal preservation.

**Figure 4:**
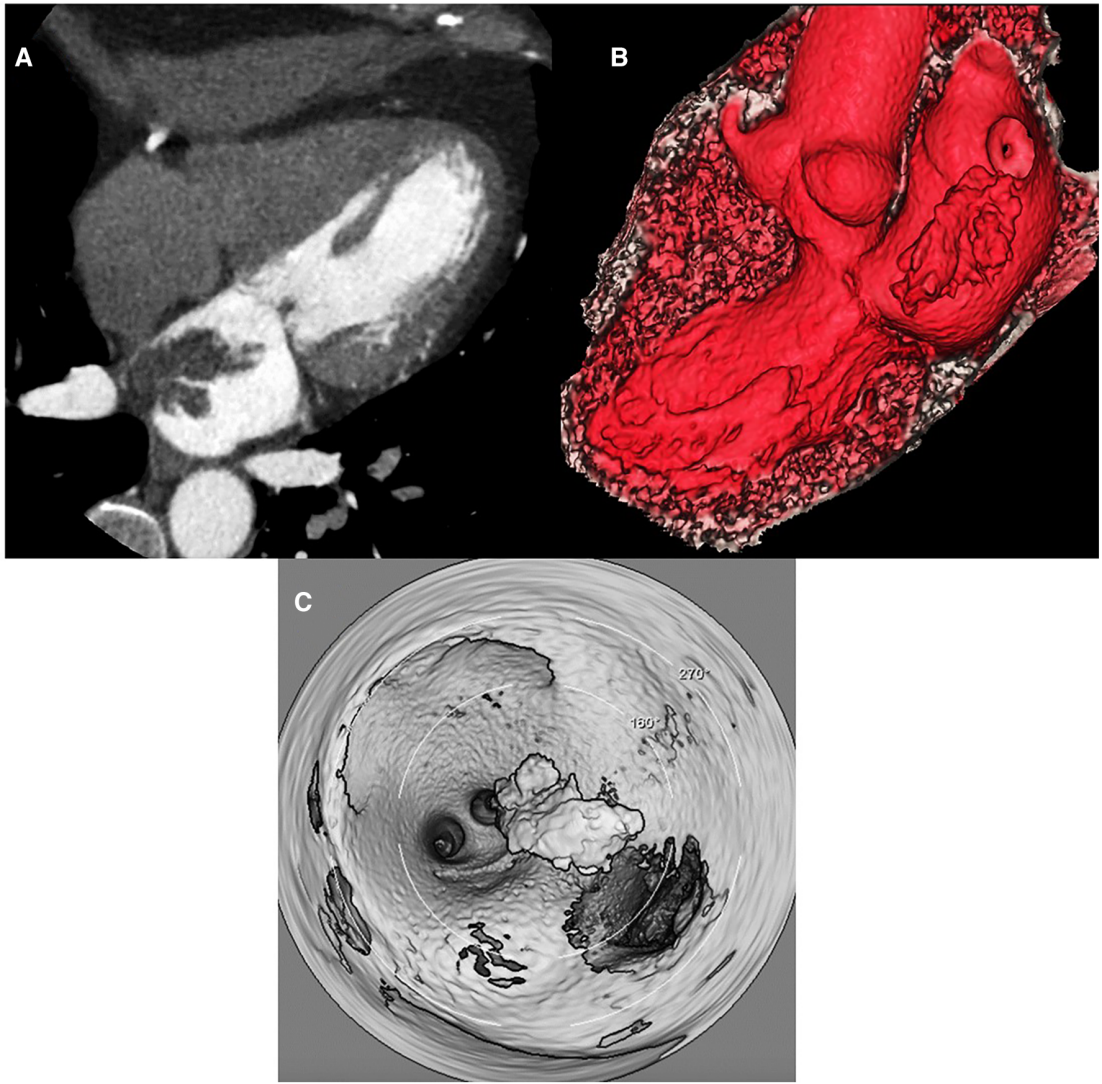
Cardiac computed tomography/3D-tools. (**A**) Cardiac contrast enhanced computed tomography horizontal long axis (4 – chambers) shows a lobulated, frond-like left atrial mass with villous borders (high risk of embolism), diameters of 39 × 39 × 20 mm, heterogeneous, isodense with punctate hypodense areas (clots), arising from the upper interatrial septum. (**B**) Oblique reformat volumen rendered 3D cardiac tomography demonstrated shows realistic anatomical relationships of left atrial mass near the ostium of the right inferior pulmonary vein. (**C**) Three-dimensional intracardiac navigation of 360 degrees revealed the dependence of the interatrial septum and the relationship with pulmonary veins of atrial mass.

The admission electrocardiogram (ECG) showed sinus rhythm associated with ST-segment elevation in leads I, II, III, aVF, V5, V6 (Figure 1A). Therefore, the initial diagnosis of an acute myocardial infarction with inferolateral ST-segment elevation (Killip-Kimball II) was made and the patient was transferred to the cardiac catheterization laboratory. Coronary angiography revealed coronary arteries without atherosclerotic lesions (Figure 2A and Supplementary Movie S1–S5) and left ventriculography exhibited apical ballooning (Figures 2B,C and Supplementary Movie S6). Hemodynamic monitoring showed elevated left ventricular (LV) end-diastolic pressure (25 mmHg) and a pressure gradient between the LV and the aorta of 24 mmHg (see Supplementary Material). Subsequently, she was transferred to the coronary care unit for management. Transthoracic echocardiography (TTE) was performed, highlighting a characteristic contractile pattern: hypokinesia/akinesia of the apical-medial segments and hyperkinesis of the basal segments, which resulted in LV systolic dysfunction (LVEF 48%, LVEDVi 61.25 ml/m^2^) (Figure 3A and Supplementary Movie S7, S8) and type II diastolic dysfunction [with elevated filling pressures (E/e' ratio 18) and left atrium dilatation (35 ml/m^2^)]. Furthermore, incidentally, a mobile, pedunculated, 51 × 18 mm long mass was found in the left atrium, adhered to the interatrial septum (Figure 3B). It should be noted that the intraventricular color Doppler was not turbulent. Finally, the strain analysis of the left ventricle confirmed the severe compromise of myocardial function at the apical-medial levels (Figure 3C). The cardiac computed tomography (CT) and its 3D tools better characterized the intracavitary mass, showing a bilobed, villous, 39 × 39 × 20 mm long tumor of low attenuation, and additionally demonstrated uninvolved pulmonary veins (Figures 4A–C and Supplementary Movie S9–S11). Because ischemic strokes are a recognized cause of TTS, a brain CT scan was performed to rule out a new acute ischemic embolic event. The most relevant finding was the sequel hypodensity at the posterior limb of the internal capsule. Chest CT scan showed signs of basal pulmonary congestion without the presence of consolidation or pleural effusion. Relevant laboratory tests retrospectively showed elevated troponin I 22.26 pg/ml (normal range <34 pg/ml), NT-proBNP was 625 pg/ml (normal range <125 pg/ml) and inflammatory markers were elevated with discrete leukocytosis (11,200 WBC/mm^3^) with no left shift and a C-reactive protein value of 78 mg/L (normal range <10 mg/L), blood and urine cultures were negative and renal and thyroid function markers were normal. All these findings allow ruling out the most common triggers of TTS, supporting the hypothesis of myxoma as a precipitant of the syndrome.

## Treatment

**Figure 5:**
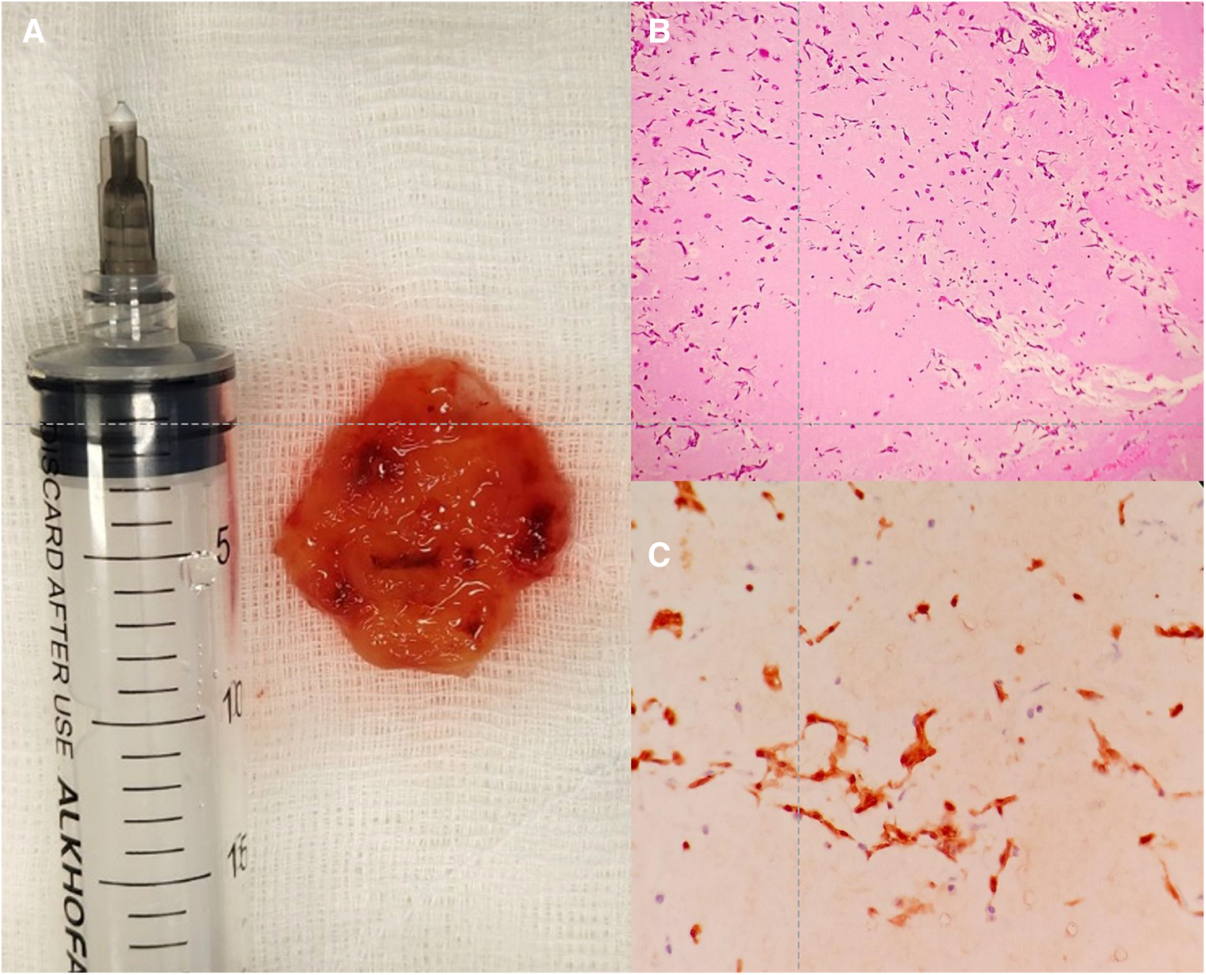
Anatomical piece and histology of the atrial mass. (**A**) Ovoid, reddish tumor, with a hairy surface, friable to the touch, approximately 4.0 cm^2^ × 3.9 cm^2^ in length. (**B**) Histology, stellate cells without atypia, immersed in vascularized myxoid tissue. HIM 4×. (**C**) Cells positive for calretinin staining, 10×.

Given the findings described, the following diagnoses were proposed: TTS complicated with LV outflow tract obstruction (LVOTO), and cardiac mass, probable left atrial myxoma. The clinical picture was managed with non-invasive ventilation (high-flow nasal cannula, FiO2 50%, 50 Lt/min), high-dose loop diuretics, and complete anticoagulation with low molecular weight heparin. Forty-eight hours after admission, pulmonary decongestion was achieved and oxygenation support was progressively withdrawn. On the fifth day, the Heart Team decided the resection of the left atrial mass due to the high risk of embolization, removing a round, reddish tumor, with a villous surface, friable to the touch and measuring approximately 4.0 cm^2^ × 3.9 cm^2^ in length (Figure 5A). The pathological anatomy confirms the diagnosis of atrial myxoma (Figure 5B). There were no significant complications in the postoperative period and the patient was discharged early and without any problems. During hospitalization, successive electrocardiograms showed the usual course of TTS (Figures 1B,C).

At 6 weeks of follow-up, the patient remains cardiovascular asymptomatic with NYHA functional class I. Control TTE ruled out recurrence of the mass and confirmed the normalization of LV segmental motility with recovery of LVEF (Supplementary Movie S12), which retrospectively supports the proposed diagnosis.

## Discussion

Over the years and with the greater availability of different diagnostic tools, TTS diagnosis has increased worldwide (1). It is estimated that this syndrome represents approximately 2% of all patients diagnosed with ACS and approximately 10% of ACS in women (2). Typically, 90% of TTS cases occur in women, with an average age between 65 and 70 years (3, 7). On the other hand, primary cardiac tumors are extraordinary, with a frequency of approximately 0.02%, corresponding to 200 tumors per million autopsies (5). These occur more frequently in women, in a ratio of 2:1, with an average age between 50 and 60 years (6). The presence of these two pathologies is extremely rare and there are only anecdotal reports of this association (4, 8, 9). In this case report, the gender and age of the patient are correlated with the epidemiology of these two entities.

Several pathophysiological mechanisms have been proposed for the development of TTS. However, there is considerable evidence that excessive sympathetic stimulation is essential for its pathogenesis (10). Despite this, the exact mechanism by which excess catecholamines precipitate myocardial stunning in different coronary territories that characterize this condition is unknown. Another fundamental characteristic of the syndrome is its relationship with stressful events as precipitants of the clinical picture (11). Various emotional or physical precipitants have been described, including cerebrovascular events and tumors. Myxomas can induce TTS through a variety of neurological (acute cerebrovascular embolism and progressive involvement of the central autonomic network through distant tumoral seeding, which can affect the cardiovascular regulation center at the CSN, generating dysregulation of the autonomic pathways and predisposing towards a more severe and persistent adrenergic discharge, which favors the development of TTS) and inflammatory mechanisms (directly triggering TTS or lowering the threshold for its development, in the context of inappropriate adrenergic discharge) (12) (CENTRAL ILLUSTRATION). Thus, we proposed atrial myxoma as the trigger of the TTS.

**Central Illustration:**
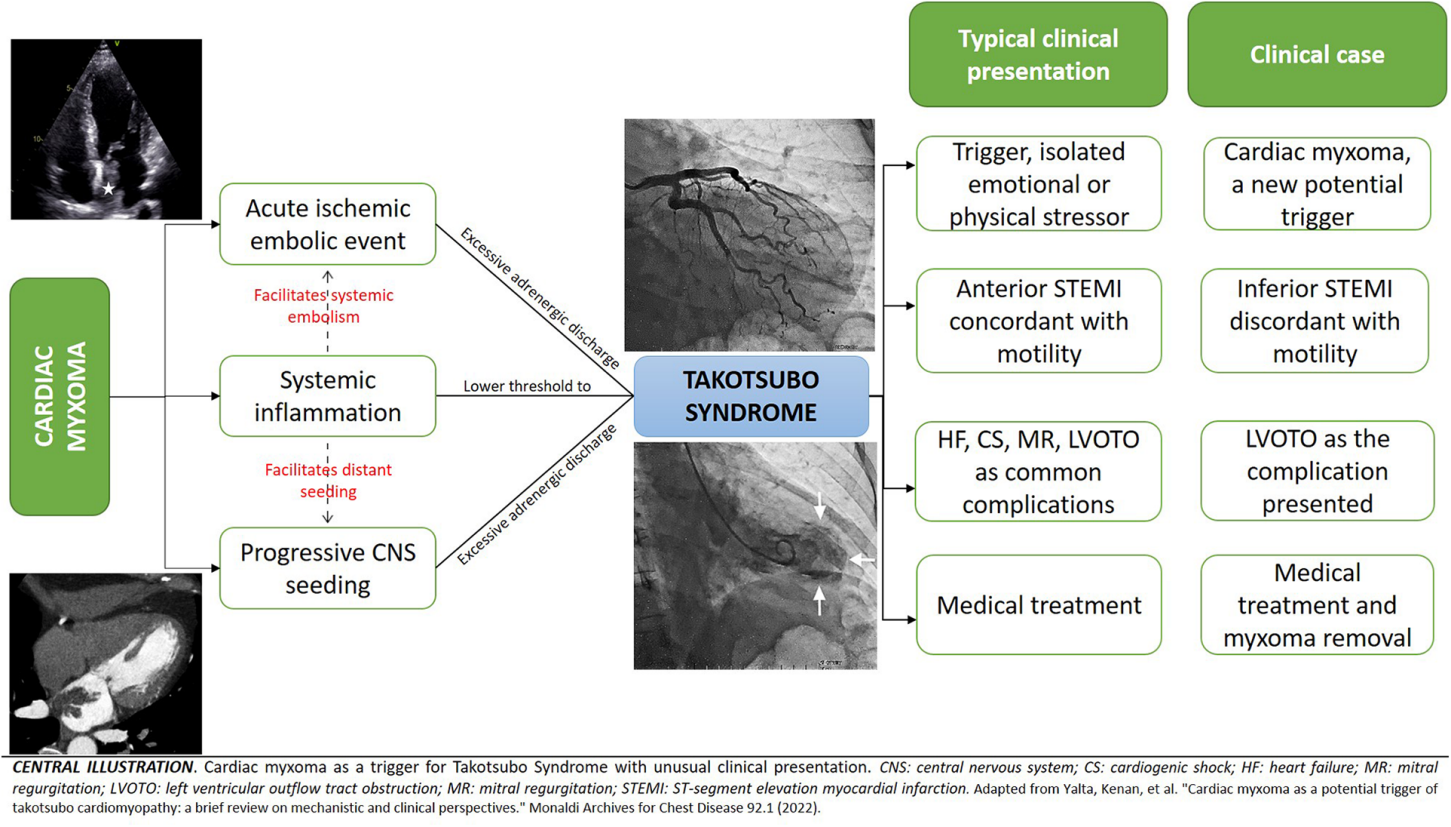


The clinical phenotype often mimics an acute coronary syndrome in terms of symptomatology, electrocardiographic changes, and cardiac biomarkers. The most common symptoms are chest pain, dyspnea, or syncope. However, the clinical manifestations of the acute stressor may predominate. From an electrocardiographic point of view, 98% of patients present with an abnormal electrocardiogram (44% have ST-segment elevation, 41% T wave inversion, and 8% ST-segment depression) (13). ST-segment usually involves the precordial and lateral leads (V2–V5), resembling an anterolateral infarction, while ST-segment elevation in inferior leads (II, III, aVF) is particularly rare (14). On the other hand, progressive inversion of T waves as well as prolongation of the QT interval are common electrocardiographic findings in TTS. The inversion of T waves usually occurs in the same leads with elevation of the ST-segment, although its distribution can be broader and deeper, while the long QT can be a substrate for the development of ventricular tachycardia and/or sudden death (10, 15). In our patient, the rarity of the trigger and the electrocardiographic findings of ST-segment elevation in the inferior leads differ from the usual presentation of TTS. However, all these electrocardiographic findings, together with motility disorders involving more than one arterial territory, the typical ventriculographic pattern, the complete recovery of motility defects at 6 weeks, and the presence of frequent complications of TTS supported our diagnosis.

Various complications during the acute phase of the disease can occur (cardiogenic shock, heart failure, LVOTO, mitral regurgitation, malignant arrhythmias, and embolic events) (15, 16). The latter is usually secondary to the formation of clots in the akinetic segment of the affected ventricle, associated with systemic inflammation and activation of the coagulation cascade (12). Despite this, there are no clear guidelines regarding the use of anticoagulation in the setting of atrial myxoma. Some studies suggest a lack of benefit of antiplatelet or anticoagulant therapy with respect to the prevention of cerebrovascular events (17), while others report favorable results with the use of anticoagulation (18). Given the mixed results and the lack of solid evidence regarding the use of these drugs, we considered that the benefit of full anticoagulation outweighed the risk, considering the short bridging time to cardiac surgery. What is more, the presence of a left atrial mass in the context of a hyperadrenergic state (with an inherent risk of atrial arrhythmias) further increases the probability of systemic embolism. Within the evaluation of the risk of systemic embolization, the use of pulsed wave tissue Doppler imaging (PW-TDI) makes it possible to determine the movement, velocity and acceleration of intracardiac masses. Those masses with a higher instantaneous velocity are associated with a greater outward force, which would increase the probability of the mass being ejected into the bloodstream. Thus, a mass peak antegrade velocity (Va) ≥ 10 cm/s has been proposed as a marker of embolization, allowing identification of patients at risk and timely decision making (19).

LVOTO occurs in around 20% of patients with TTS, usually due to hyperkinesis of the basal segments together with an increased interventricular septum thickness (due to underlying myocardial edema) produce high-flow velocity in a narrowed LVOT, resulting in a Venturi effect (suction effect) in the mitral valve apparatus with septal anterior motion (SAM), which generates dynamic obstruction at the level of the outflow tract. LVOTO can improve with the use of beta-blockers, however, these are contraindicated in patients with acute heart failure, hypotension, and bradycardia. Likewise, the use of nitrates in the context of acute heart failure to reduce afterload can worsen the pressure gradient and should therefore be avoided. This clearly reflects the complexity of managing a patient with TTS and its complications. The clinical consequences of TTS in our patient were mainly acute heart failure and the increased gradient of the left ventricular outflow tract. Preload optimization with loop diuretics and non-invasive ventilation were sufficient to compensate the patient and allow early surgical intervention.

## Conclusions

The presence of atrial myxoma and TTS is extraordinarily rare. Different pathways can explain a cause/effect relationship between them, and the clinical presentation will depend on the underlying pathophysiological events. Coronary angiography with left ventriculography is the gold standard for the diagnosis of TTS, while multimodal imaging detects and characterizes cardiac masses. In the treatment of these concurrent pathologies, the initial objective is to stabilize the clinical picture of TTS and subsequently remove the tumor mass.

## Data Availability

The original contributions presented in the study are included in the article/Supplementary Material, further inquiries can be directed to the corresponding author.

## Supplementary material

Supplementary Figure

Left ventricular and aortic hemodynamic curves.

Supplementary Movie S1

LAO 45°, CRA 20° – Diagnostic Coronary Angiography – Normal right coronary circulation.

Supplementary Movie S2

RAO 30°, CRA 0° – Diagnostic Coronary Angiography – Normal right coronary circulation.

Supplementary Movie S3

RAO 25°, CAU 25° – Diagnostic Coronary Angiography – Normal left coronary circulation.

Supplementary Movie S4

RAO 15°, CRA 35° – Diagnostic Coronary Angiography – Normal left coronary circulation.

Supplementary Movie S5

LAO 40°, CAU 25° – Diagnostic Coronary Angiography – Normal left coronary circulation.

Supplementary Movie S6

RAO view – Contrast left ventriculography.

Supplementary Movie S7

TTE, 4 chambers, apical akinesia and myxoma are seen in the left atrium.

Supplementary Movie S8

TTE, 2 chambers, apical akinesia and myxoma are seen in the left atrium.

Supplementary Movie S9

Cardiac contrast enhanced computed tomography horizontal long axis (4 – chambers).

Supplementary Movie S10

Oblique reformat volumen rendered 3D cardiac tomography.

Supplementary Movie S11

Three-dimensional intracardiac navigation of 360 degrees.

Supplementary Movie S12

TTE, 4 chambers, absence of myxoma recurrence and LVEF recovery.
