# Supplementary figures and images for: Takotsubo syndrome and atrial myxoma—identifying a new trigger: a case report
Source: Front Cardiovasc Med. 2024 Feb 13;11:1323492. doi: 10.3389/fcvm.2024.1323492 (PMC10897023; doi:10.3389/fcvm.2024.1323492)

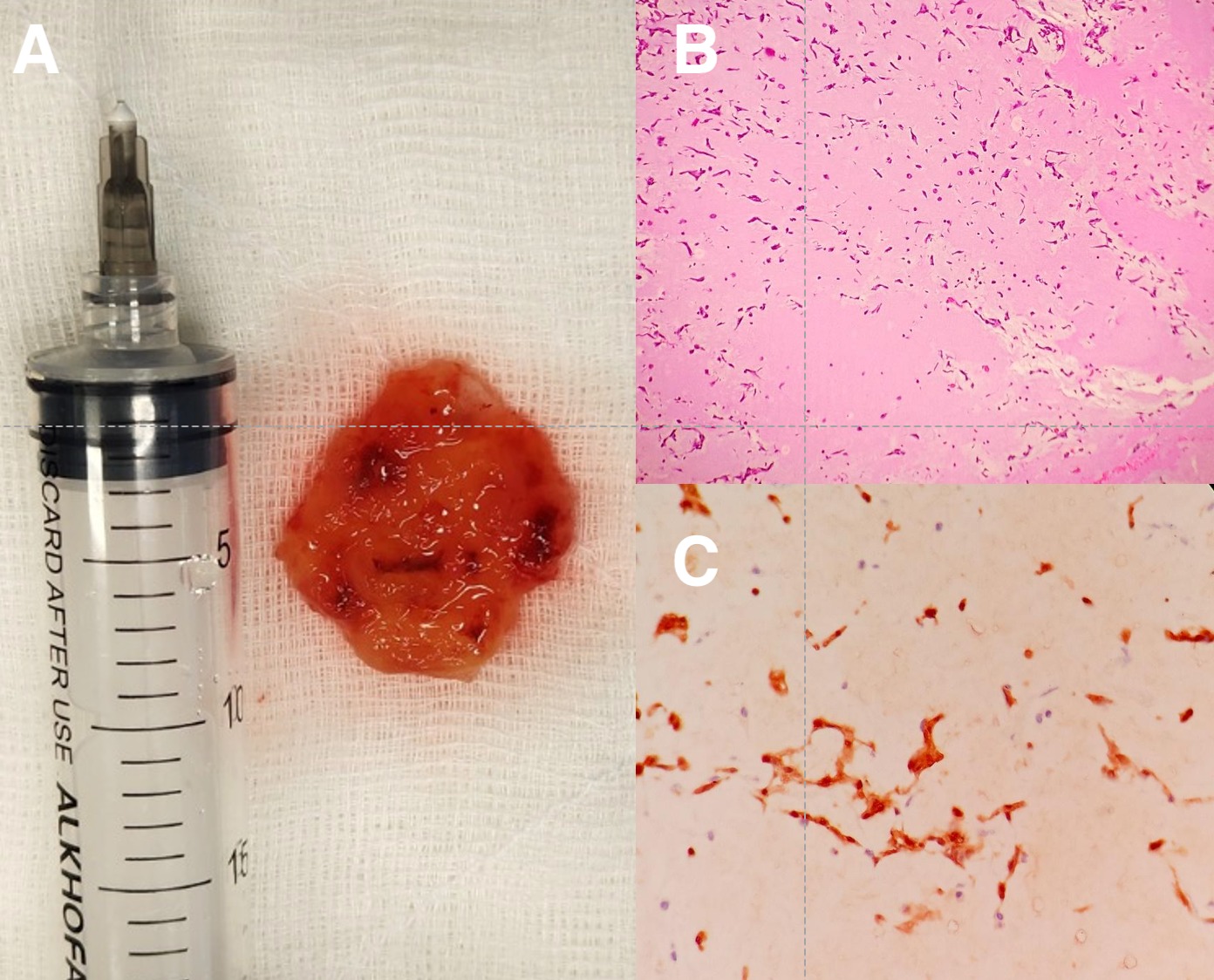

Supplement: Supplementary file 13 [file Image1.jpeg]

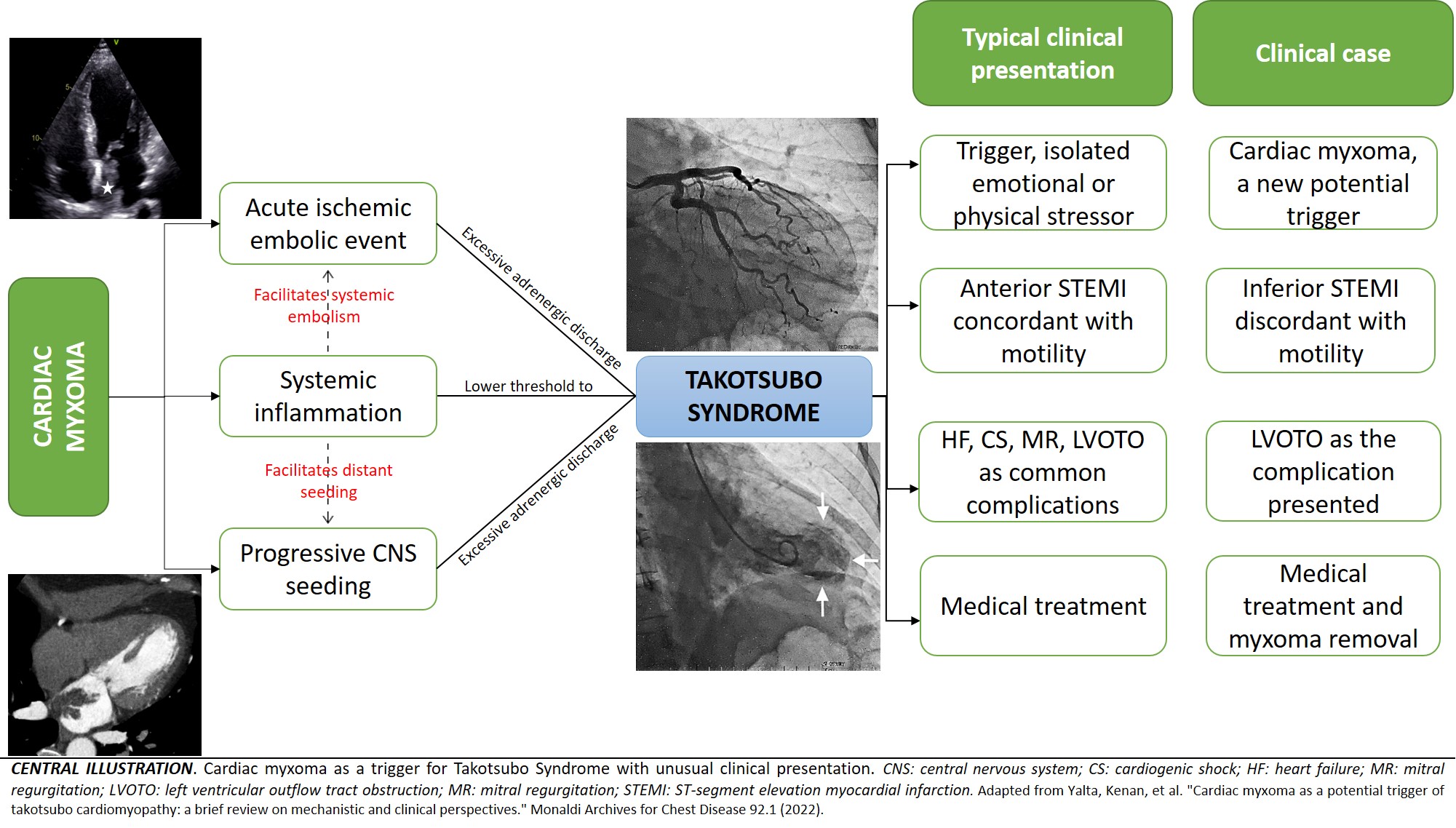

Supplement: Supplementary file 14 [file Image2.jpeg]

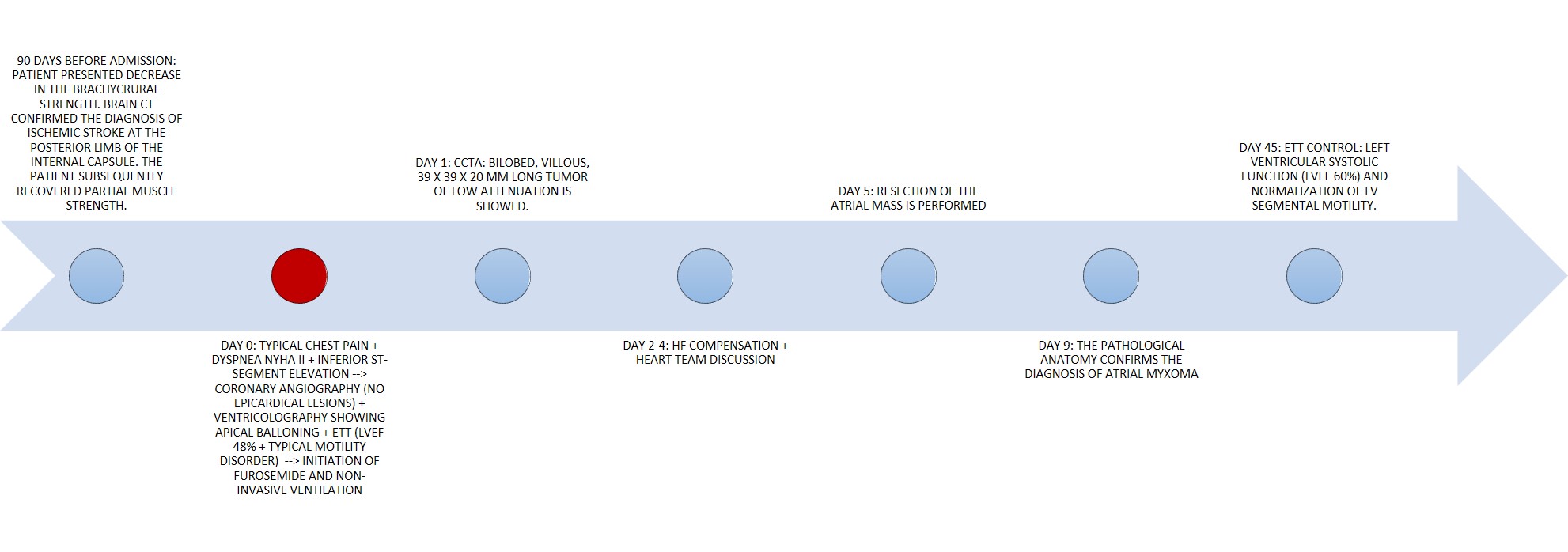

Supplement: Supplementary file 15 [file Image3.jpeg]

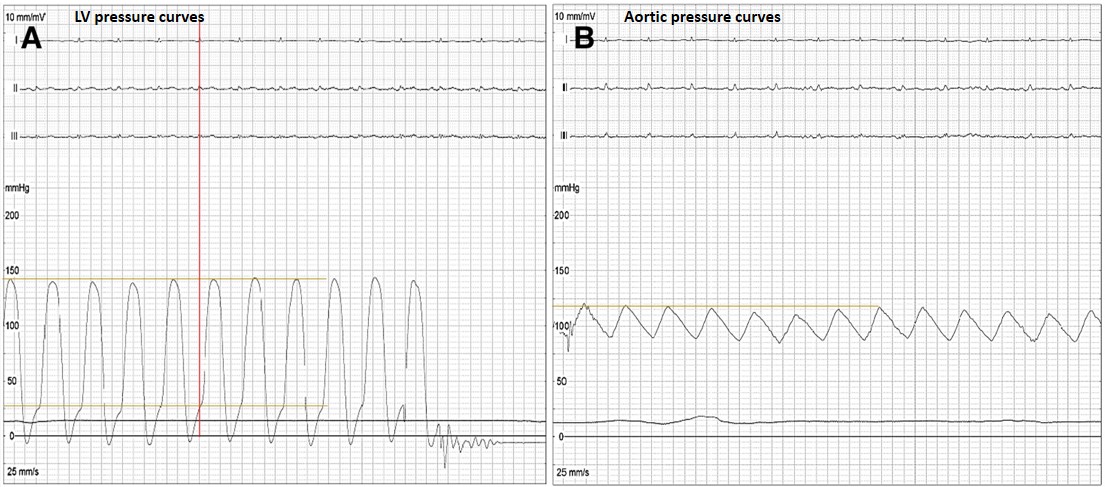

Supplement: Supplementary file 16 [file Image4.jpeg]
